# Cross-jurisdictional data access processes and coordination in two countries: key learnings and innovative approaches.

**DOI:** 10.23889/ijpds.v7i3.2033

**Published:** 2022-08-25

**Authors:** Marie-Chantal Ethier, Juliana Wu, Carina Marshall, Felicity Flack

**Affiliations:** 1 Health Data Research Network (HDRN) Canada; 2 Canadian Institute for Health Information (CIHI); 3 Population Health Research Network (PHRN) Australia

## Objectives

Access to cross-jurisdictional data can be beneficial to researchers but challenges exist in accessing data from different legal jurisdictions and policy environments. Two national networks in Australia and Canada will share their respective approaches to supporting the discovery of, and coordinating applications and approvals for, cross-jurisdictional data access.

## Approach

Collaboration between these two networks in the last year have allowed members to gain a better understanding of the approaches that have been put in place to support the coordination of cross-jurisdictional data applications. Through regular inter-network discussion and joint workshops, we have gained insight into the similarities, differences and challenges faced by each and identified common goals for continued shared learnings.

## Results

Similarities and differences exist between the approach and processes each network has implemented to coordinate applications for cross-jurisdictional data. In both countries, a single point of contact model has been key in coordinating data requests centrally. Whereas in one country, virtual access to linked data is provided centrally, different legal and policy environments across jurisdictions in another country are barriers to accessing data in one location. Each network has developed processes and tools to meet researcher needs while respecting local requirements and are turning to innovative strategies. For example, one network is developing infrastructure to support analysis of data in a distributed way, and both groups are creating centralized tools to streamline the data discovery and access processes to improve researcher experience.

## Conclusion

The coordination of applications for cross-jurisdictional data access by two national networks has streamlined researcher access, but challenges exist to fully meet researcher needs for cross-jurisdictional analyses. The continued cross-network collaboration will allow for shared learning opportunities and development of innovative solutions to tackle challenges related to cross-jurisdictional data access.

